# Unraveling dimethylformamide-induced neutrophilic differentiation in HL-60 cells: A proteomic and functional comparison with dimethyl sulfoxide

**DOI:** 10.1371/journal.pone.0348783

**Published:** 2026-05-13

**Authors:** Othman Eldalal, Yasser Tabana, Dinesh Babu, Newton H. Tran, Steven Lockhart, Joshua Kranrod, John M. Seubert, Lusine Tonoyan, Richard P. Fahlman, Arno G. Siraki

**Affiliations:** 1 Faculty of Pharmacy & Pharmaceutical Sciences, College of Health Sciences, University of Alberta, Edmonton, Canada; 2 Department of Drug Technology, Faculty of Medical Technology, Derna, Libya; 3 Department of Biochemistry, Faculty of Medicine and Dentistry, University of Alberta, Edmonton, Canada; 4 Li Ka Shing Institute of Virology, University of Alberta, Edmonton, Canada; University of New Hampshire, UNITED STATES OF AMERICA

## Abstract

The differentiation of HL-60 cells into neutrophil-like cells is widely used to study neutrophil functions, yet no comprehensive proteomic analysis has been conducted on dimethylformamide (DMF)-induced differentiation. This study provides the first detailed proteomic characterization of DMF-differentiated (df)-HL-60 cells, demonstrating its distinct molecular and functional profiles compared to the well-established dimethyl sulfoxide (DMSO)-df-HL-60 cell model. HL-60 cells were differentiated using 1.25% DMSO or 70 mM DMF for five days. Cell proliferation, granulocytic differentiation (CD11b expression), superoxide anion production, myeloperoxidase (MPO) protein expression and enzymatic activity, and neutrophil extracellular trap (NET) formation were evaluated. Proteomic profiling was performed using LC-MS/MS, followed by gene ontology and pathway enrichment analysis to identify key molecular changes associated with differentiation. DMF-df-HL-60 cells maintained higher proliferation rates than DMSO-df-HL-60 cells. Both agents successfully induced granulocytic differentiation, with DMSO producing greater CD11b expression. Functionally, both differentiation methods enhanced superoxide anion production, but DMF-df-HL-60 cells generated distinct superoxide radical spectra when evaluated with EPR spectroscopy. MPO protein expression and activity were significantly reduced in both differentiation models, indicating a transition to a mature neutrophil-like phenotype. Proteomic analysis revealed that neutrophil degranulation was the most significantly enriched pathway in DMF-df-HL-60 cells, alongside pathways involved in oxidant production and receptor tyrosine kinase signaling. Furthermore, S100 calcium-binding protein A9 (S100A9) abundance was significantly higher in DMF-df-HL-60 cells, suggesting a novel role of DMF in modulating neutrophil differentiation. DMF-df-HL-60 cells also showed activation of MAPK, Ras, and Rap1 signaling pathways, similar to the DMSO-df-HL-60 cell model, which is crucial for differentiation and immune responses. DMF-df-HL-60 cells generated more NETs than the DMSO-df-HL-60 cell model with phorbol myristate acetate. This study emphasizes the importance of selecting the appropriate differentiation model to accurately mimic neutrophil biology and highlights DMF’s unique role in neutrophil differentiation, providing novel insights into differentiation-induced functional adaptations.

## 1. Introduction

Neutrophils are an essential element of the innate immune system, as they are the body’s primary defense against infections and play a vital role in inflammatory responses. They are known for their ability to rapidly migrate to infection sites, undergo degranulation, produce oxidants, and release neutrophil extracellular traps (NETs) to kill pathogens [1]. However, chronic inflammatory conditions and autoimmune disorders are impacted by dysregulated neutrophil activity [2]. Understanding the differentiation and functional activation of neutrophils is essential for developing therapeutic strategies to regulate their activity in pathological conditions. Despite the pivotal role of neutrophils in both physiological and pathological conditions, studying their behavior *in vitro* remains challenging. Neutrophils have a short *in vitro* lifespan, undergo apoptosis within 6–12 hours post-isolation, and lack the ability to be cryopreserved or expanded. They require fresh isolation from human donors, which introduces variability and reproducibility challenges, complicating long-term studies [3–5]. Additionally, neutrophils are challenging to genetically manipulate, limiting the ability to induce mutations or modify gene expression [5]. These constraints have compelled researchers to explore different models for investigating neutrophil functions.

The HL-60 cell line, originating from a 36-year-old woman with promyelocytic leukemia, is a frequently used model for studying neutrophil functions [3]. In contrast to primary neutrophils, HL-60 cells can be easily cultured *in vitro* and genetically modified to introduce mutations or alter gene expression [6]. Under specific conditions, HL-60 cells can be differentiated into neutrophil-like cells, displaying segmented nuclei and azurophilic granules, making them a suitable model for studying neutrophil functions [6,7].

The commonly used reagents to induce neutrophil-like differentiation of HL-60 cells include all-trans retinoic acid (ATRA), dibutyryl cyclic adenosine monophosphate (dbcAMP), dimethyl sulfoxide (DMSO), and dimethylformamide (DMF) [4,8–10]. Although dbcAMP modulates cAMP signaling pathways and ATRA regulates transcription through retinoic acid receptors [4], the mechanisms by which DMF and DMSO induce differentiation are less understood. Previous studies have reported DMSO-induced differentiation of HL-60 cells into neutrophils with considerable alterations in protein composition and upregulation of key proteins, such as β2-integrin (CD11b/CD18), CD35, and the glycosylphosphatidylinositol-anchored protein GPI-80, which are linked to neutrophil phagocytosis [11]. Gene expression analysis has also shown successive alterations during the differentiation process of HL-60 cells, characterized by an early upregulation of cell cycle inhibitors and transcription factors, followed by genes linked to apoptosis, RNA processing, and marking the acquisition of a mature neutrophil phenotype [12]. However, there have been few findings that show how this affects HL-60 cell differentiation by altering membrane permeability and impacting nucleic acid and protein synthesis, which could be crucial to molecular mechanisms driving neutrophil maturation and functionality [4,10–12]. DMSO is produced naturally through the oxidation of dimethylsulfide and is widely used as an industrial solvent. Moreover, it has various pharmacologic actions, including anti-inflammatory effects, local analgesia, bacteriostatic properties, diuretic effects, enzyme inhibition, and vasodilation [6,13]. Several studies have revealed that DMSO effectively differentiates HL-60 cells into neutrophil-like cells [6,13]. Similarly, DMF is primarily involved in many chemical syntheses, such as the production of synthetic leather [14]. It has also been characterized as a promising differentiating agent for HL-60 cells into neutrophil-like cells [15]. Although less thoroughly studied than DMSO, DMF has recently gained significant attention in pharmacological research due to its beneficial properties, including antioxidant effects that reduce cerebral ischemia damage, particularly in diabetic rats [16]. DMF also has the ability to inhibit fungal growth and aflatoxin production in addition to its potential to modulate cell proliferation and apoptosis in breast cancer [16–18]. While DMSO is used as a standard differentiation agent, evaluating DMF’s advantages and differences is crucial for understanding its potential as an alternative. Although DMF is known as an effective inducer of neutrophil-like differentiation in HL-60 cells and has been reported as a suitable model for studying neutrophil extracellular trap (NET) formation and the resultant cell death, NETosis [8], the proteomic profile of this differentiation model has not been studied yet. In contrast, DMSO-induced differentiation has been extensively examined at both the molecular and functional levels [8,19]. The present study aims to characterize the proteomic profile of DMF-differentiated (df)-HL-60 cells and to perform a direct, side-by-side functional and proteomic comparison with the DMSO-df-HL-60 cell model under commonly used differentiation conditions. This approach reveals distinct and shared key proteins and pathways associated with neutrophil functions, including oxidative burst and immune responses. In addition, the suitability of both the differentiation models for studying NETosis is investigated.

## 2. Materials and methods

### 2.1. Materials

HL-60 human promyelocytic leukemia cells (#CCL-240) were purchased from ATCC, Manassas, VA, via Cedarlane Labs, Burlington, ON. Transfer-Blot® SD semi-dry transfer cell and 4X Laemmli buffer were acquired from Bio-Rad (Hercules, CA), while the BLUelf prestained protein ladder was purchased from FroggaBio (North York, ON). Immobilon Western chemiluminescent HRP substrate and anti-human myeloperoxidase (MPO) rabbit polyclonal antibody were obtained from EMD Millipore (Billerica, MA), and anti-rabbit IgG, HRP-linked antibody was purchased from Cell Signaling Technology (Whitby, ON). Roswell Park Memorial Institute (RPMI-1640) medium and Hanks’ Balanced Salt Solution (HBSS) were acquired from Gibco. Nitrotetrazolium blue chloride (NBT) was obtained from Abcam (Cat: ab146262) DMSO, DMF, diphenyleneiodonium chloride (DPI), diethylenetriaminepentaacetic acid (DTPA), hydrogen peroxide (H_2_O_2_), protease inhibitor cocktail, guaiacol, phorbol 12-myristate 13-acetate (PMA), and bovine superoxide dismutase (SOD) were all purchased from Sigma-Aldrich Canada (Oakville, ON), and were of the highest grade available. Sodium phosphate buffer (PB, 0.1 M, pH 7.4) was prepared in-house and treated with 100 μM DTPA (metal-chelating agent) to prevent metal-catalyzed auto-oxidation [20]. The 5,5-dimethyl-1-pyrroline-N-oxide (DMPO) and the Pierce™BCA protein assay kit were obtained from Thermo Scientific (Rockford, IL). APC-Cy7 mouse anti-human CD11b was obtained from BD Pharmingen (Cat: 560914). 12 x 75 mm Round Bottom Polystyrene Test Tubes were purchased from BD Falcon (Ref# 352054). Phosphate-buffered saline (PBS; pH 7.4, 1 × , without Ca²⁺ and Mg²⁺) was obtained from Gibco (Cat. No. 10010–023). 35 mm uncoated, gamma-irradiated glass-bottom dishes (No. 1.0) were purchased from MatTek (Cat:P35G-1.0-20-C). Sytox Green was obtained from Thermo Fisher Scientific (Cat: 7020), and Sytox Red was purchased from Thermo Fisher Scientific (Cat: S34859). Calcium ionophore A23187 was obtained from Abcam (ab235979), and MPO-FITC was purchased from Abcam (ab11729). Poly-L-lysine solution was purchased from Sigma-Aldrich (Cat: P8920). Bovine serum albumin (BSA) and Triton X-100 were obtained from Sigma-Aldrich (BSA: (Lot. No. SLBC8307V); Triton X-100: Lot No. MKBJ3318V). Phenol red-free RPMI 1640 (1×) medium was purchased from Gibco (Lot No. 2993927).

### 2.2. Cell differentiation and growth rate assessment

HL-60 cells were cultured in RPMI-1640 medium supplemented with 10% (v/v) fetal bovine serum (FBS) and 1% penicillin and streptomycin. The cells were maintained in a 5% CO₂ humidified incubator at 37°C. The cells were passaged every three days, and only those that had been passaged no more than 15 times were used for experimentation. To induce differentiation of HL-60 cells into granulocyte-like cells, the cells were seeded at a density of 2 × 10⁵ cells/mL in complete RPMI-1640 in a six-well plate. The differentiation was achieved by treating HL-60 cells with either 1.25% DMSO or 70 mM DMF for 5 days. The concentrations of the differentiating agents and the incubation period were selected based on the previously reported, well-established HL-60 differentiation protocols [8,21–26]. On the first and third days of differentiation, additional RPMI-1640 medium containing the same concentrations of DMSO or DMF was added to the cultures to maintain the treatment conditions. Throughout the differentiation period, cell counts were performed daily using trypan blue dye and a cell counting machine (The Countess™ II FL Automated Cell Counter) to monitor cell viability and growth.

### 2.3. Assessment of differentiation of HL-60 cells (df-HL-60 cells)

#### 2.3.1. Measurement of neutrophil surface marker expression.

The differences in the expression of neutrophil surface markers between the untreated and differentiation-inducing agent-treated HL-60 (df-HL-60) cells were assessed by flow cytometry. On the final day of the differentiation protocol, the cells were washed three times and resuspended at a concentration of 1 x 10^6^ cells in 100 µL of PBS containing 2% FBS. The cells were stained with APC-conjugated CD11b antibody and incubated at 4°C for 30 minutes. After incubation, the cells were washed and fixed with 4% paraformaldehyde at 4°C for 30 minutes. Following fixation, the cell suspension was washed to remove any cell aggregates and resuspended in PBS containing 2% FBS. The samples were acquired using an LSRFortessa flow cytometer (Becton Dickinson). The marker expression was evaluated by analyzing the fluorescence intensity histogram of the APC-conjugated CD11b antibody. A minimum of 10,000 events were recorded at the lowest cell suspension rate. Further analysis of the experimental data was performed using FlowJo software (version 10).

#### 2.3.2. Measurement of superoxide anion production.

NBT-reducing activity was performed to assess the ability of df-HL-60 to produce superoxide anion (O_2_^•-^). NADPH oxidase (NOX) forms O_2_^•-^ from NADPH and oxygen. The O_2_^•-^ generated can reduce NBT to a formazan, which can be detected by UV-visible spectrophotometry (SpectraMax M5, US). This assay was performed as previously described [27,28] with a slight modification. In brief, 1 × 10⁶ cells were prepared from both HL-60 and df-HL-60 groups, which were differentiated by DMSO or DMF. The cells were washed three times by centrifuging at 300 × g for 7 minutes, and then the cell pellet was resuspended in HBSS. For the negative control, 10 µM DPI was added to one set of samples from each group and incubated on a shaker at 37°C for 1 hour. To stimulate ROS production, 1 µM PMA and 1 mg/mL NBT were added simultaneously to all groups and incubated in the dark for an hour. Subsequently, the samples were centrifuged, and the supernatant was carefully discarded. The pellet was washed with methanol and then centrifuged again at 200 × g for 5 minutes. A solubilization reagent was prepared by mixing 1 M NaOH, DMSO, and isopropanol. This mixture was added to each group to release the precipitated formazan from the cells. The mixture was vortexed thoroughly and the solubilized sample was transferred into a 96-well plate. The solubilization reagent alone was added to separate wells without cells as a blank. Finally, the absorbance was measured at 620 nm to quantify ROS production. The degree of differentiation was assayed by the ability of the cells to reduce NBT into insoluble formazan upon stimulation with PMA.

#### 2.3.3. Characterization of radical production.

The superoxide radical anion was detected using electron paramagnetic resonance (EPR) spectroscopy, as previously described, with slight modifications [29]. This technique used the spin trap agent, DMPO, which reacts with short-lived radicals to form a stable, measurable signal. Briefly, 1 × 10⁶ cells from both the HL-60 and df-HL-60 groups treated with either DMSO or DMF, were washed three times, and then suspended in 100 µL HBSS. 100 mM DMPO was added to the cells, followed by treatment with 1 µM PMA to stimulate the respiratory burst. The reaction mixture was incubated at 37°C, with 300 rpm rotation on a shaker for 15 minutes. The reaction mixture was then transferred into separate 50 μL microcapillary tubes and inserted simultaneously into a 3 mm EPR tube, which was then placed in the resonator. EPR spectra were obtained using continuous wave EPR with a Bruker Elexsys E-500 spectrometer equipped with a SHQ cavity (Billerica, MA) with the following parameters: Center field = 3505 G, sweep width = 100 G, field modulation = 1 G, microwave frequency = 9.8 GHz, microwave power = 20 mW, and sweep time = 120 s.

#### 2.3.4. Western blot analysis of MPO levels in df-HL-60 cells.

Previous studies have reported a decrease in MPO mRNA expression during the differentiation of HL-60 cells, indicating a shift towards a neutrophil phenotype [30]. However, to our knowledge, changes in MPO protein levels during the differentiation process have not been reported. To investigate this further, protein lysates of HL-60 and DMSO/DMF-df-HL-60 cells were prepared using PBS containing 1% SDS and 1% protease inhibitor cocktail. The samples were incubated for 45 minutes, followed by sonication for 1 minute to ensure complete cell lysis and homogenization. The mixtures were then centrifuged at 15,000 rpm for 20 minutes at 4°C, and the supernatant was collected. Protein concentration was determined using the pierce ^TM^ BCA Protein Assay Kit according to the manufacturer’s instructions. 5 µg of protein lysates were mixed with 4x Laemmli buffer and run on the same gel for electrophoretic separation, then transferred onto nitrocellulose membranes using a semi-dry method. The membranes were blocked with a blocking buffer consisting of 5% non-fat skim milk in Tris-buffered saline with 0.1% Tween-20 (TBST) for 1 hour at room temperature. The membranes were then incubated overnight at 4°C with primary anti-MPO rabbit polyclonal antibody (1:4000) in blocking buffer. After washing three times with TBST, the membranes were incubated with a secondary goat anti-rabbit IgG, HRP-linked antibody (1:5000) for 1 hour at room temperature. Following MPO detection, the membranes were again washed three times with TBST and then incubated overnight at room temperature with β-actin HRP-conjugated antibody (1:5000) as a loading control. The membranes were washed three times with TBST after each step of immunoblotting. Immunoblots were visualized with Immobilon Western Chemiluminescent HRP substrate using an ImageQuant LAS 4000 mini luminescent image analyzer (GE Healthcare, Pittsburgh, PA). The band intensities were quantified using ImageJ laboratory software version 6 (Bio-Rad Laboratories; CA, USA) and expressed as mean ± SD (n = 3).

#### 2.3.5. UV-Visible spectroscopic analysis of MPO activity.

MPO peroxidation activity was measured as previously described [31]. In this assay, guaiacol was used as the substrate, and hydrogen peroxide (H₂O₂) was added as the cofactor. MPO catalyzes the oxidation of guaiacol in the presence of H₂O₂ (24), leading to the formation of a brown-colored product called tetra guaiacol, which was monitored. The quantity of tetra guaiacol produced directly corresponds to the MPO peroxidase activity in the tested sample. Briefly, 1 × 10⁶ cells from HL-60 and df-HL-60 groups treated with DMSO or DMF were washed two times and carefully suspended in ice-cold 0.1 M phosphate buffer with 100 µM DTPA. Following that, 10 mM guaiacol was added to the cell suspension. To initiate the reaction, 400 µM of H_2_O_2_ was added to the samples, and the absorbance readings were measured immediately using a UV-visible spectrophotometer (SpectraMax M5, US). The kinetic activity of MPO was then monitored over 20 minutes at a wavelength of 470 nm, with readings taken every 30 seconds.

### 2.4. Assessment of neutrophil extracellular trap (NET) formation

#### 2.4.1. Quantification of NET formation.

After assessing ROS production, NET release was evaluated as another key neutrophil effector function using Sytox Green plate assay (12). Sytox Green is a membrane-impermeant DNA-binding dye, used to quantify extracellular DNA release as a surrogate readout of NETosis [32]. The differentiated HL-60 cells (5 × 10⁵ cells/mL) were suspended in serum-free, phenol red-free RPMI medium and seeded into a 96-well black plate, then allowed to settle for 30 minutes at 37°C in a 5% CO₂ incubator. Where indicated, the NOX inhibitor diphenyleneiodonium (DPI) (20 µM) was added and cells were incubated for 1 hour at 37°C. The unstimulated cells were included as negative controls. NETosis was induced using the NOX-dependent agonist PMA (500 nM) or the NOX-independent agonist calcium ionophore A23187 (4 µM), followed by incubation for 6 hours at 37°C. Sytox Green (0.5 µM) was then added, and plates were incubated for 10 min in the dark. Fluorescence was measured using a plate reader (excitation 504 nm, emission 523 nm), and NETosis was calculated based on the Sytox Green fluorescence (relative fluorescence units, RFU).

#### 2.4.2. Visualization of NET  formation.

The NETs were visualized using fluorescence microscopy [8]. The differentiated cells were seeded in 35 mm glass-bottom dishes coated with 0.01% poly-L-lysine. Cells were allowed to settle for 30 min, and then NET formation was stimulated with PMA, the most frequently used stimulus with a 100% success rate for inducing NETosis [33]. After a 6hour stimulation, samples were ﬁxed with 4%paraformaldehyde for 20 minutes, permeabilized with 0.1% Triton X-100 for 60 seconds, and blocked with 1% BSA. The samples were incubated overnight at 4°C with anti-MPO conjugated with FITC (1:500, λ_Ex/Em_ = 488/519 nm) followed by washing three times. DNA was stained with Sytox Red (400 nM, λ_Ex/Em_ = 640/658 nm) for 10 minutes in the dark, followed by washing three times. All images were analyzed using a Zeiss Axio Observer Z1 inverted epifluorescence microscope with a 40 X/1.3NA oil DIC objective lens. The image system Zeiss ZENsoftware was used for analysis.

### 2.5. Gel-LC-MS/MS

**Fig 1:**
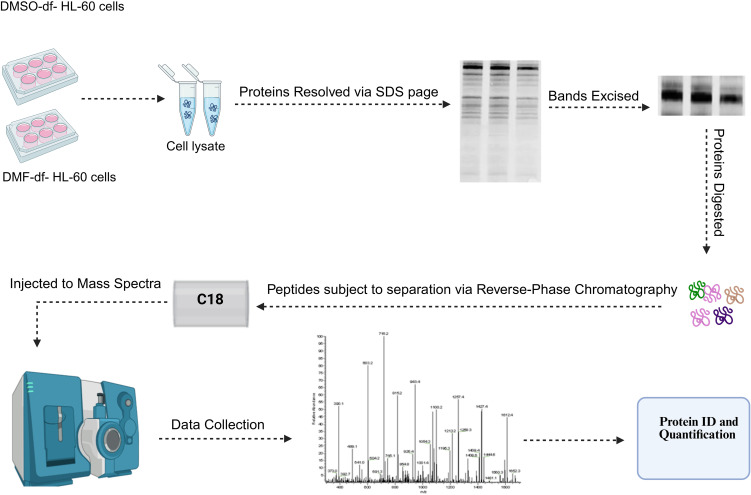
Workflow for preparing cell samples for proteomic studies.

For the initial shotgun proteomic analysis, untreated HL-60 (Control), DMSO- and DMF-df-HL-60 cells were selected. Cell lysates were resolved on a 10% SDS-PAGE gel and visualized using Coomassie staining. Each gel lane was divided into small slices, and proteins within each slice underwent in-gel tryptic digestion as outlined in previously established protocols [34]. The resulting peptide mixtures were vacuum-dried and reconstituted in solvent A (5% acetonitrile (ACN) in 0.2% formic acid) for subsequent LC-MS/MS analysis. Peptide identification was conducted using a Thermo Scientific EASY-nL 1000 system coupled to a Q-Exactive Hybrid Quadrupole-Orbitrap Mass Spectrometer. The LC-MS/MS analysis employed a 75-minute gradient with operational parameters similar to those described in earlier studies [35]. The workflow for this method is illustrated in Fig 1.

#### 2.5.1. MS/MS data analysis.

The raw MS/MS spectral data for each sample were combined and analyzed using Proteome Discoverer 1.4.1.14 (Thermo Fisher Scientific). The database search was performed against the reviewed, non-redundant Homo sapiens proteome obtained from UniProt, with parameters based on established methods. The identified proteins and their respective extracted ion chromatograms (EICs), reflecting protein abundance, are listed in S1 Table [36].

#### 2.5.2. Data analysis.

Gene Ontology (GO) analysis for the identified protein groups was performed using the Metascape platform (https://metascape.org) [37] (PMI, a comprehensive tool for functional enrichment and pathway analysis). For the visualization of normalized and scaled data, heatmaps were generated using the Heatmapper tool (http://www.heatmapper.ca) [38], enabling hierarchical clustering and effective graphical representation. We utilized the “Proteomaps” tool (available at www.proteomaps.net) to analyze the quantitative composition of pathways and cellular processes that are particularly susceptible to changes [39].

#### 2.5.3. Quantification and statistical analysis.

Data are presented as mean ± SD. Following LC-MS analysis, protein abundance was quantified using the extracted ion chromatograms (EICs) and normalized to the total ion current (TIC). Statistical comparisons among the three groups were performed using one-way analysis of variance (ANOVA), with a P-value < 0.05 considered statistically.

## 3. Results

### 3.1. Differences in HL-60 cell growth and survival following DMSO or DMF treatment

Cell proliferation was evaluated in untreated, DMSO-df-HL-60 cells, and DMF-df-HL-60 cells, starting with an initial seeding density of 200,000 cells per group for a period of 0–5 days. On day 3, HL-60 cells treated with DMSO exhibited a significant reduction in proliferation, with a mean cell count of 475,000, which was significantly lower than both DMF-treated cells (791,667 ± 73; ^#^ *P* < 0.05) and untreated HL-60 cells (680,333 ± 47; *P < 0.05). No statistically significant difference was observed between the untreated and DMF-treated HL-60 cells at this time point. By day 5, untreated HL-60 cells continued to proliferate, reaching a mean count of 1,246,667 ± 38. In contrast, cells treated with DMSO showed a marked and highly significant inhibition of proliferation, with a reduced mean count of 379,333 ± 23 (***P < 0.0001) as compared to untreated cells. The DMF-treated cells reached a plateau at 817,000 ± 70, a value significantly lower than the untreated group (**P < 0.01), but not significantly different from their own day 3 cell count (791,667 ± 73), suggesting an arrest of cell proliferation and a likely shift towards differentiation.

**Fig 2:**
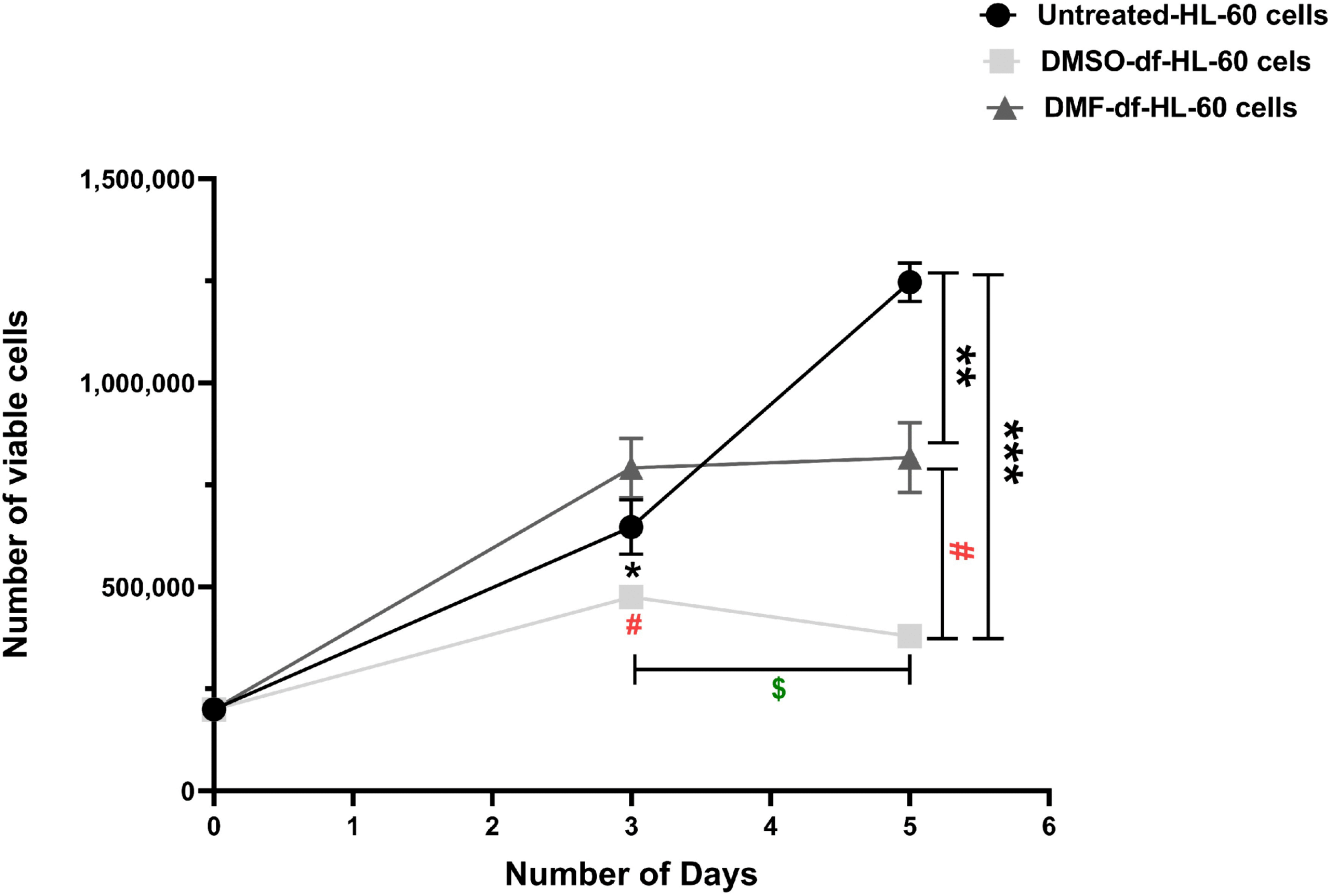
The impact of HL-60 cell proliferation by DMSO or DMF treatment. HL-60 cells were differentiated to neutrophil-like cells by DMSO or DMF. HL-60 cells (2 × 10⁵/mL) were seeded into a T-75 flask and treated with 1.25% DMSO or 70 mM DMF by addition of fresh agent on days 0, 1, and 3 for a total differentiation period of five days Cell viability was assessed using the trypan blue dye exclusion assay. (*, **, and ***) indicates comparison with untreated HL-60 cells (* *P* < 0.05, **P ≤ 0.01, *** *P* < 0.0001). (^#^) indicates comparison between DMSO-df- and DMF-df-HL-60 cells at the same time point (^#^ P < 0.05). (^$^) indicates comparison between Day 3 and Day 5 within the same group (^$^ *P* < 0.05). The growth curve showed rapid growth in untreated HL-60 cells compared to differentiated cells following DMSO and DMF treatments. Both DMSO and DMF showed no significant proliferation after day 3, with a significant decrease in DMSO-df-HL-60 cells. Data are presented as mean ± SD of viable cells (n = 3).

Importantly, the DMSO-df-HL-60 cells showed a substantially lower cell number than the DMF-df-HL-60 cells at day 5 (^#^ *P* < 0.05). This suggests that DMSO not only induces differentiation but may also induce cell death. Furthermore, within the DMSO group, a significant decline in cell count was observed between day 3 (475,000 ± 26) and day 5 (379,333 ± 23, ^$^ *P* < 0.05), indicating a progressive inhibition of cell proliferation or potential cell death following prolonged DMSO exposure Fig 2.

### 3.2. Changes in the expression of CD11b in HL-60 cells under the influence of DMSO or DMF

**Fig 3:**
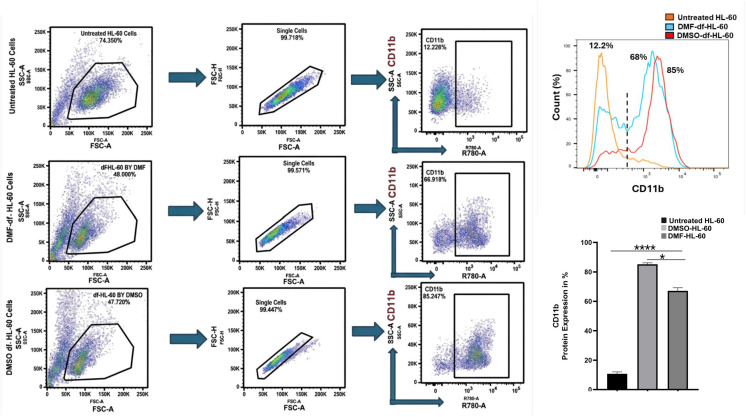
Quantification of CD11b expression in untreated and differentiated HL-60 cells. **(A)** Flow cytometry gating strategy: Cells were first gated based on FSC/SSC profiles to identify the main cell population, followed by gating for single cells and CD11b stained cells. **(B)** The histograms show percentages of CD11b-positive cells. DMSO-df-HL-60 cells demonstrated the highest expression of CD11b compared to DMF-df-HL-60 cells and untreated cells. **(C)** Bar graph summarizing the percentage of CD11b-positive cells across the three groups. Data are represented as mean ± SD (n = 3). Statistical analysis was performed using one-way ANOVA with significance defined as: (****P *<* 0.0001, *P < 0.05).

To evaluate the differentiation of HL-60 cells toward granulocytes, the expression of CD11b, a distinct surface marker of granulocyte differentiation, was assessed among untreated HL-60, DMSO-, and DMF-df-HL-60 cells. We observed changes in CD11b expression in HL-60 cells under the influence of the differentiation agents (Fig 3). CD11b expression was significantly higher in differentiated cells compared to untreated HL-60 cells (*P* < 0.001). DMSO exhibited the highest CD11b expression, reaching approximately 85%, whereas DMF increased CD11b expression to approximately 68% (*P* < 0.05). In contrast, untreated HL-60 cells exhibited the lowest CD11b expression, at about 10%. These findings indicate that treatment with DMSO or DMF promotes HL-60 differentiation toward granulocytes.

### 3.3. DMSO- or DMF-treated HL-60 cells show enhanced oxidative burst capacity

**Fig 4:**
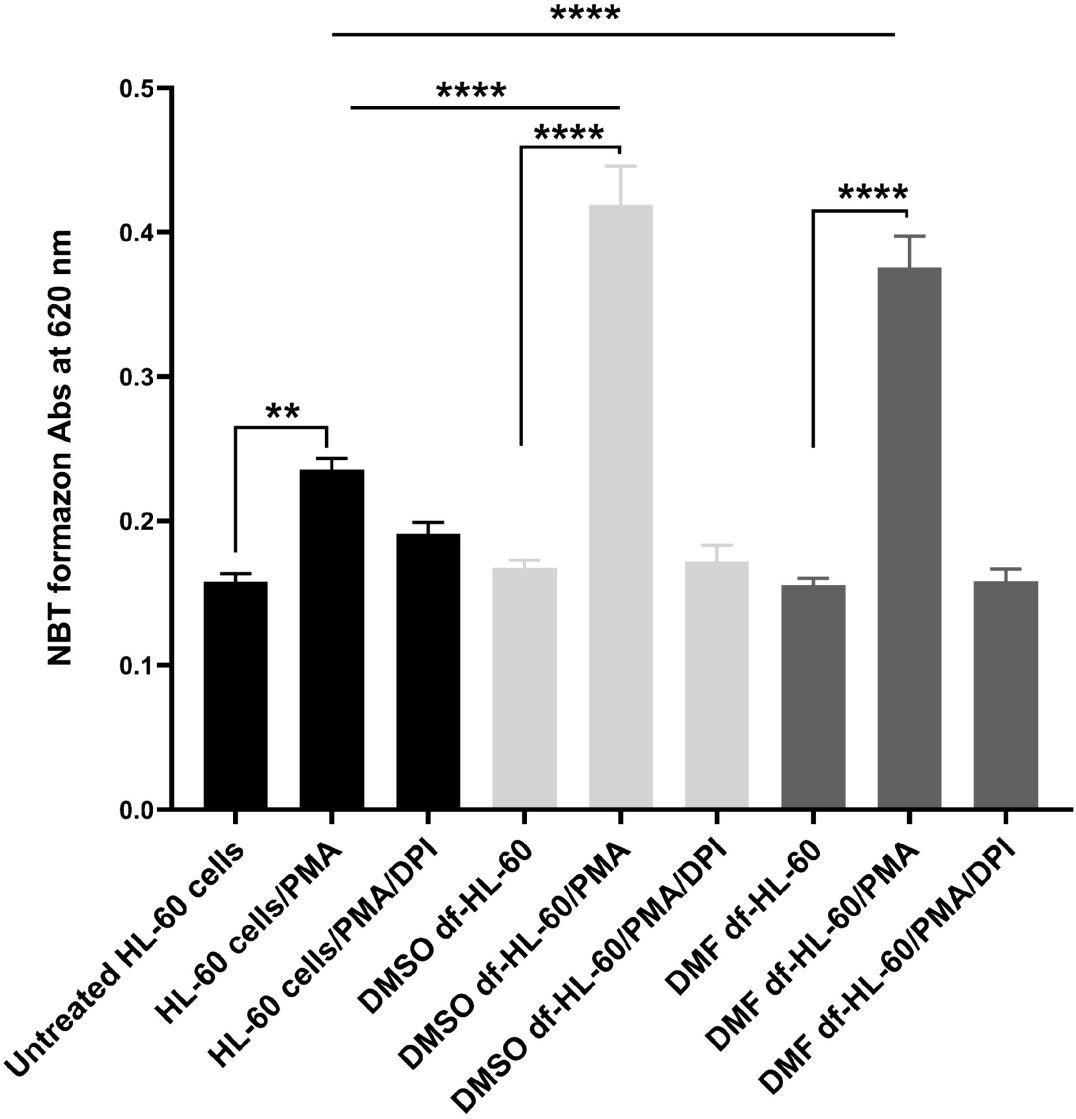
Measurement of superoxide anion production in undifferentiated and differentiated HL-60 cells. The absorbance units represent the relative levels of ROS production. $ indicates comparison of PMA-stimulated DMSO- or DMF-df-HL-60 cells with PMA-stimulated undifferentiated HL-60 cells. * indicates comparison between between each PMA-stimulated group and its corresponding untreated group. The bars represent the mean ± SD of three independent experiments (n = 3). Statistical analysis was performed using one-way ANOVA with significance defined as follows: (**P < 0.01, ****P < 0.0001, ^$$$$^ *P*< 0.0001).

The oxidative burst capacity of HL-60 cells was evaluated using the NBT assay in both untreated cells and cells differentiated with either DMSO or DMF. Cells were stimulated with PMA, a protein kinase C activator, followed by incubation with NBT. PMA stimulation induces assembly and activation of the NADPH oxidase (NOX) complex, resulting in the production of superoxide anion (O_2_^●─^). During this process, the soluble and colorless NBT is reduced via O_2_^●─^ produced by active cells within the phagosome, turning into insoluble blue formazan crystals that serve as an indicator of oxidative burst activity [27,28]. The results revealed a significant increase in NBT reduction in df-HL-60 cells compared to untreated HL-60 cells (Fig 4). Both DMSO- and DMF-df-HL-60 cells showed increased levels of radical production (P < 0.0001) as compared to untreated HL-60 cells, indicated by a strong dark blue precipitate, reflecting robust O_2_^●─^ generation and confirming effective differentiation into neutrophil-like cells. In contrast, following PMA stimulation, untreated HL-60 cells exhibited minimal NBT reduction, resulting in a lighter yellowish color, indicative of low ROS production. The addition of DPI abolished the intensity of the NBT signals in both differentiated groups, supporting the conclusion that a considerable portion of the detected radicals was O_2_^●─^.

### 3.4. EPR analysis highlights superoxide radical formation in DMF-treated HL-60 cells

**Fig 5:**
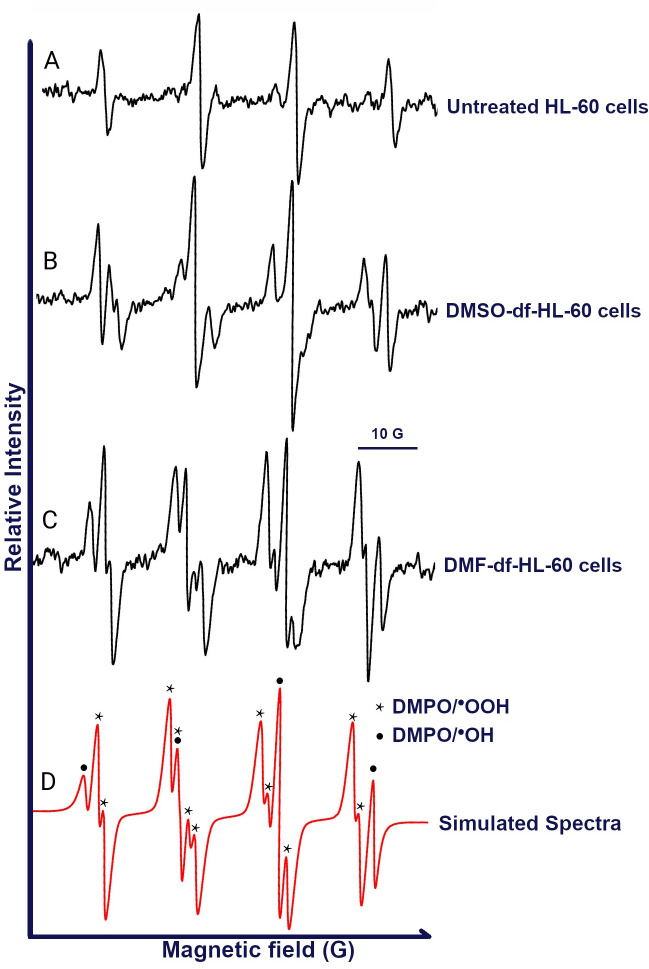
Identification of superoxide anion radical (O₂^•^⁻) from differentiated HL-60 cells. The reactions were performed using untreated HL-60, DMSO-, and DMF-df-HL-60 cells. Cells were treated with 100 mM DMPO in 100 µL HBSS and stimulated with 1 µM PMA to induce the respiratory burst. The spectra were obtained from 1 × 10⁶ cells after 15 minutes of gentle mixing (300 rpm) at 37 °C for untreated HL-60 **(A)**, DMSO-df-HL-60 **(B)**, and DMF-df-HL-60 (C) cells. Hyperfine splittings corresponding to superoxide (DMPO/^•^OOH) (marked by stars) and DMPO/^•^OH (marked by dots) were identified and shown on the simulated spectrum **(D)**. The reaction mixtures were transferred to 3 x 50 µL micro-capillary tubes and placed in a 3 mm EPR tube for spectral acquisition. In D, the simulated spectrum (correlation of r = 0.97) of DMF-df-HL-60 cells (in C) is shown. It is a composite spectrum of DMPO/^•^OH (hyperfine splitting constants: *a*^N^ = *a*^H^ = 14.9 G) and DMPO/^•^OOH (hyperfine splitting constants: *a*^N^ = 14.2 G, *a*^Hβ^ = 11.1 G, *a*^Hγ^ = 1.3 **G)**.

In this study, we used a novel tool to confirm the differentiation of HL-60 by characterizing the superoxide production using EPR spectroscopy. EPR with DMPO trapping was used to assess the O_2_^●─^ radical identification in DMSO- and DMF-df-HL-60 cells (Fig 5). Our observations showed that DMSO- and DMF-df-HL-60 cells demonstrated higher levels of radicals relative to untreated HL-60 cells. More importantly, in DMF-df-HL-60 cells, distinct hyperfine splittings correspond to O_2_^●─^ radical (DMPO/^•^OOH). The DMPO/^•^OH adducts were observed as background, indicating the presence of both superoxide anion and hydroxyl radicals.

### 3.5. Differentiation-induced changes in MPO activity in HL-60 cells

**Fig 6:**
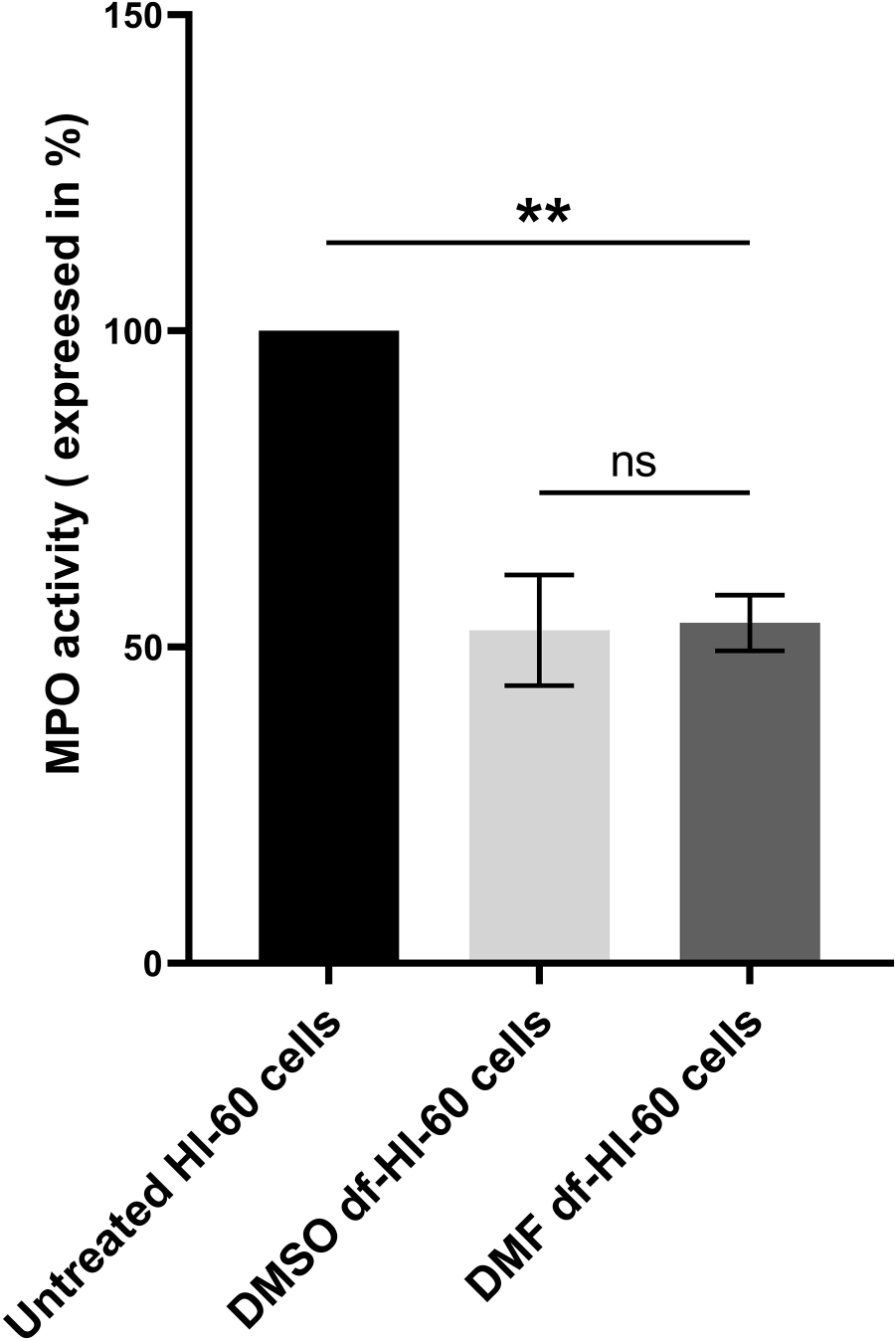
Myeloperoxidase (MPO) activity in untreated HL-60, DMSO-df-HL-60, and DMF-df-HL-60 cells. The 1-minute time point was selected for comparison among the groups to represent MPO activity. The results are expressed as a percentage, with undifferentiated HL-60 cells set as the reference (100%). Statistical analysis was performed using one-way ANOVA. The data show that MPO activity significantly decreased in both DMSO- and DMF-df-HL-60 groups by around 50% compared to undifferentiated HL-60 cells (**P < 0.01). The bars represent the mean ± SD of three independent experiments (n = 3).

MPO is a key biomarker of neutrophils, as it plays a central role in producing oxidants and free radical products during immune responses [40]. So, MPO activity was measured in HL-60 and df-HL-60 cells treated with DMSO or DMF using a guaiacol oxidation assay. Our data showed a significant reduction in MPO activity in df-HL-60 groups compared to untreated HL-60 cells. The rate of tetra-guaiacol formation, as monitored by UV-Vis spectroscopy at 470 nm over 20 minutes, was 50% lower in the differentiated cells, highlighting the decrease in MPO activity upon differentiation, as shown in Fig 6. This decline in MPO activity reflects the shift from an immature myeloid state in HL-60 cells to a more mature neutrophil-like phenotype in df-HL-60 cells

### 3.6. MPO expression declines in a time-dependent manner during HL-60 cells differentiation

**Fig 7:**
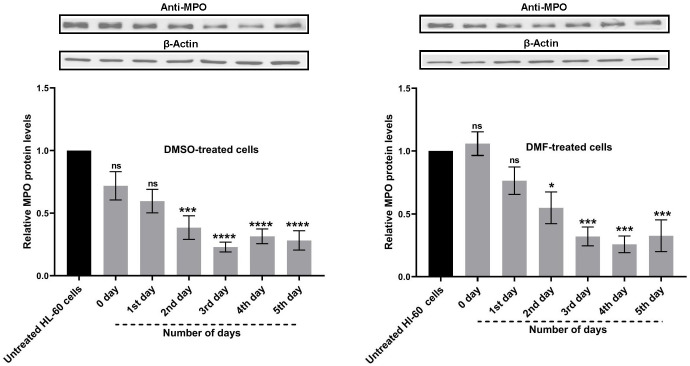
Differentiated HL-60 cells reveal a stepwise decrease in MPO expression over five days. MPO expression in HL-60 cells differentiated with DMSO (left) or DMF (right) over a five-day period. Untreated HL-60 cells were used as the baseline control, while the 0-day treated group refers to cells treated with either DMSO or DMF for 15 minutes before lysis. Data were expressed as mean ± SD, with triplicate experiments (n = 3).). Statistical analysis was performed using one-way ANOVA with significance defined as(*P ≤ 0.05, ***P ≤ 0.001, ****P ≤ 0.0001).

To investigate the changes in MPO protein expression during HL-60 differentiation, immunoblot analysis was conducted on untreated HL-60, DMSO-, and DMF-df-HL-60 cells over a five-day period. As shown in Fig 7, MPO expression progressively decreased in both treatments, with significant reductions beginning on the second day. In DMF-df-HL-60 cells, MPO expression began to decrease on day two  (*P = 0.0214) and significantly further on day three (***P = 0.0009) and continued with the same expression through days four and five. In DMSO-df-HL-60 cells, however, MPO levels showed a marked decrease by day two compared to the DMF group (***P = 0.0003), with further reductions observed on days four and five (****P *<* 0.0001). β-actin was used as a loading control to confirm equal protein loading across all lanes, supporting the reliability of these findings.

### 3.7. DMF effectively differentiated HL-60 cells into neutrophil-like cells suitable for NETosis studies

**Fig 8:**
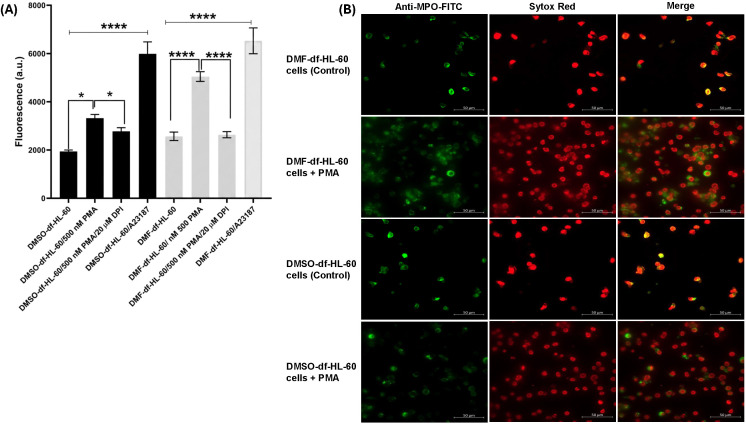
Comparison of NET formation in DMSO- or DMF-differentiated HL-60 cells. **(A)** NET formation was quantified using a Sytox Green plate reader assay after differentiation by DMSO or DMF. Differentiated HL-60 cells were left unstimulated (control) or stimulated with PMA (500 nM) (NOX-dependent) or A23187 (4 µM) (NOX-independent) for 6 **hour.** Where indicated, cells were pre-treated with diphenyleneiodonium (DPI, 20 µM) for 1 hour prior to stimulation. NETosis was reported as Sytox Green fluorescence (RFU). Data are presented as mean ± SEM from three independent experiments (n = 3).. Statistical analysis was performed using one-way ANOVA with significance defined as (*P < 0.05 and ****P < 0.0001). **(B)** Representative fluorescence microscopy images showing NET formation in DMSO- or DMF-differentiated HL-60 cells. Cells were left untreated (control) or stimulated with PMA (500 nM) for 6 hour, fixed, and stained for MPO (FITC) and DNA (Sytox Red). Scale bar: 50 µm, representative image (n = 3).

NET- release was quantified using the Sytox Green assay in DMSO- and DMF-df- HL-60 cells following stimulation with PMA (NOX-dependent pathway) or A223187 (NOX-independent pathway).As shown in Fig 8 A, PMA stimulation resulted in a significant increase in Sytox Green fluorescence in DMSO-df- HL-60 cells (*p < 0.05), whereas a markedly stronger response was observed in DMF-df- HL-60 cells (****p < 0.0001) compared with their respective unstimulated controls. Similarly, stimulation with A23187 significantly increased Sytox Green fluorescence in both differentiation models, with a highly significant increase observed in both DMSO- and DMF-df- HL-60 cells (****p < 0.0001). Under both PMA- and A23187-stimulated conditions, DMF- df-HL-60 cells exhibited significantly higher Sytox Green fluorescence than DMSO- df- HL-60 cells.

Fluorescence microscopy was performed using PMA-stimulated cells and untreated controls as representative conditions to visually confirm NET-like structures detected by the quantitative Sytox Green plate reader assay. Cells were stained with an anti-MPO antibody conjugated to FITC (green) to visualize extracellular MPO and with Sytox Red to label extracellular DNA. As shown in Fig 8B, DMF- df-HL-60 cells formed more NET-like structures following PMA stimulation, characterized by extracellular DNA co-localizing with MPO staining. In contrast, minimal extracellular staining was observed in untreated control cells.

### 3.8. Identification of proteins of differential abundance among the control, DMSO- or DMF-treated HL-60 cells

**Fig 9:**
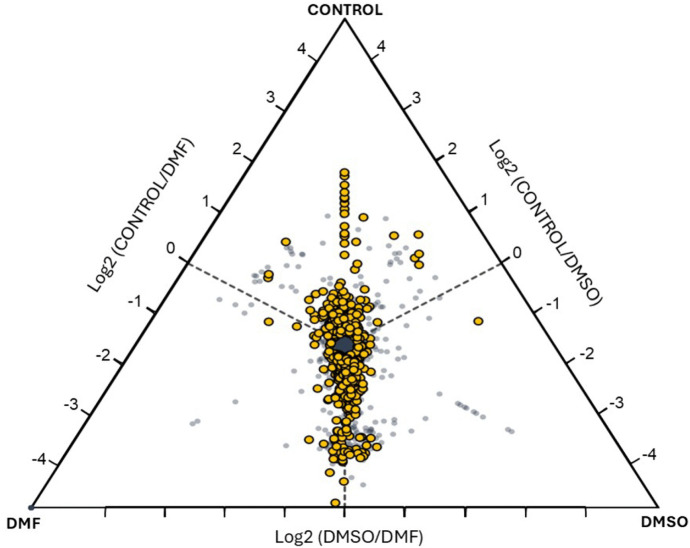
Visualization of protein expression reveals proteomic similarity between DMSO- and DMF-treated cells. A three-way tri-plot was used to visualize the differences in protein abundance across control, DMSO, and DMF -treated samples. The log2 ratio of normalized protein abundance was plotted to highlight the variations between the three groups. In this visualization, the proteins with higher abundance in one group relative to the others are positioned closer to the corresponding vertex. Significantly abundant proteins (*P* < 0.05) are shown by yellow circles.

The ratios of the average relative extracted ion intensities for each protein were compared across the three experimental groups: untreated HL-60 (Control), DMSO- and DMF-df-HL-60 cells [35]. The extracted ion intensities for each protein are the sum of ion intensities for the peptides observed and quantified for each protein (this is the numerical intensity used for all the analysis) [41]. In Fig 9, the clustering of data points toward the center of the plot indicates that the majority of proteins have similar average ion intensities across all groups. However, the proteins with higher intensities in a specific group are positioned closer to the apex corresponding to that group. To account for potential bias in average ion intensities caused by technical variability or true biological differences among individual samples, a one-way ANOVA was conducted to identify proteins showing statistically significant differences among the three groups. The proteins that met the statistical threshold (*p* < 0.05) are highlighted as yellow circles in the tri-plot. While the applied threshold may result in some false positives due to multiple testing, the proteins identified warrant further investigation to confirm their significance.

Overall, the data reveal that most proteins are shared between the DMSO and DMF groups, with fewer proteins shared with the control group. This suggests a closer proteomic similarity between the DMSO- and DMF-treated samples compared to the control, potentially reflecting shared molecular pathways or responses under similar experimental conditions.

### 3.9. Functional enrichment analysis

**Fig 10:**
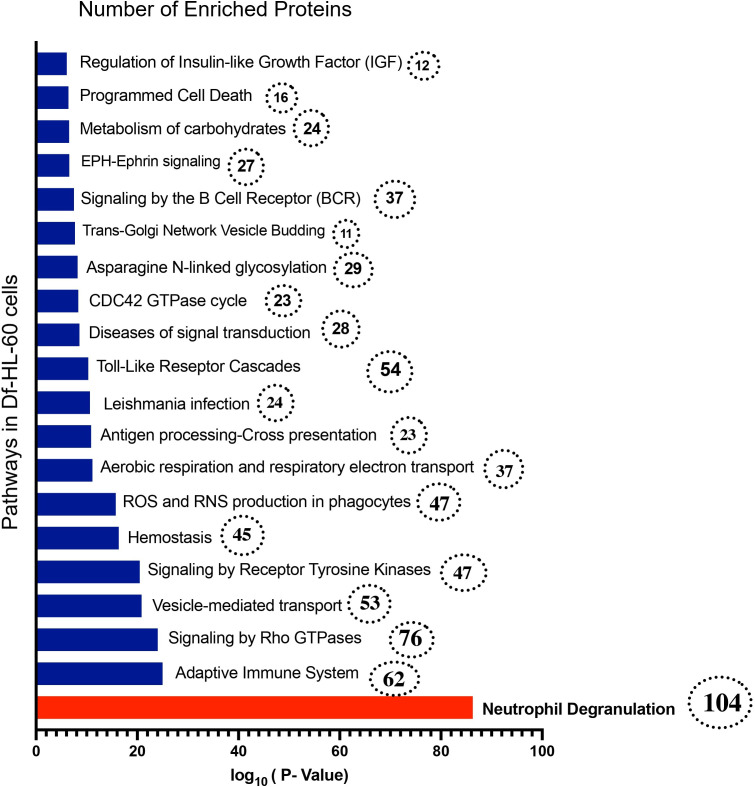
Enriched gene ontology pathways are based on the proteomic analysis of differentiated HL-60 cells treated with DMSO or DMF. Gene ontology analysis was performed on unique proteins from each group. The number of proteins from gene ontology are shown in dotted circles. A detailed list of proteins and gene ontology analysis are provided in S1 Table..

As previously observed, most proteins are similarly expressed between the DMSO- and DMF-df-HL-60 cells groups. To further analyze their functional relevance, we performed functional enrichment analysis on the shared proteins using Metascape. This analysis clustered the functions of proteins upregulated in differentiated HL-60 cells treated with both DMSO and DMF. The results revealed enrichment in pathways critical for neutrophil functionality and differentiation. Among the most significantly enriched processes were neutrophil degranulation, signaling by Rho GTPases, and adaptive immune system pathways (Fig 10). Additionally, processes such as reactive oxygen species (ROS) and reactive nitrogen species (RNS) in Phagocytes, signaling by receptor tyrosine kinases, and the Toll-like receptor (TLR) cascade were also upregulated. These findings underscore the pivotal roles of these pathways in driving neutrophil differentiation and maturation, highlighting the functional transformation of HL-60 cells into neutrophil-like cells. Since neutrophil degranulation was identified as the most enriched pathway, we further analyzed the normalized and scaled (z-score) abundances of proteins, summarized in hierarchical clustering heatmaps.

**Fig 11:**
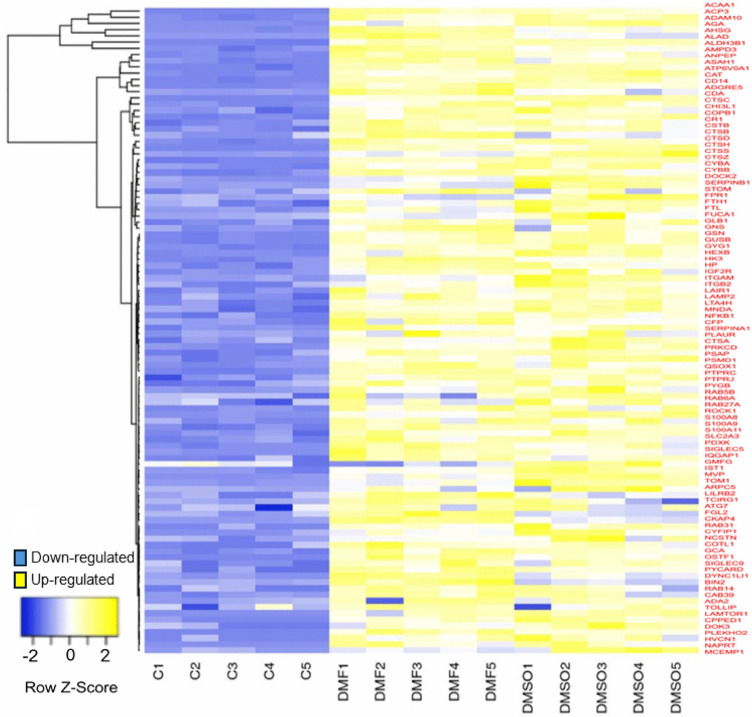
Heatmap of the genes associated with neutrophil degranulation during HL-60 differentiation. The differential expression of genes across untreated HL-60 cells (C1-C5), differentiated cells treated with DMF (DMF1-DMF5) and DMSO (DMSO1-DMSO5). Yellow indicates upregulated proteins, while blue indicates downregulated proteins.

As shown in the heatmap (Fig 11), which specifically highlights genes involved in neutrophil degranulation, these genes are significantly upregulated in differentiated HL-60 cells treated with either DMF or DMSO compared to untreated HL-60 cells (controls) (C1-C5). This upregulation is visually evident as a clear shift from blue (low relative expression in the untreated control HL-60 cells) to yellow (high relative expression in the differentiated groups), reflecting activation of genes involved in neutrophil degranulation following DMF- or DMSO-induced differentiation. Notable upregulation of key proteins associated with granule exocytosis, vesicle trafficking, and proteolytic activity was observed. A detailed list of proteins contributing to neutrophil degranulation is provided in S1 Table.

**Fig 12:**
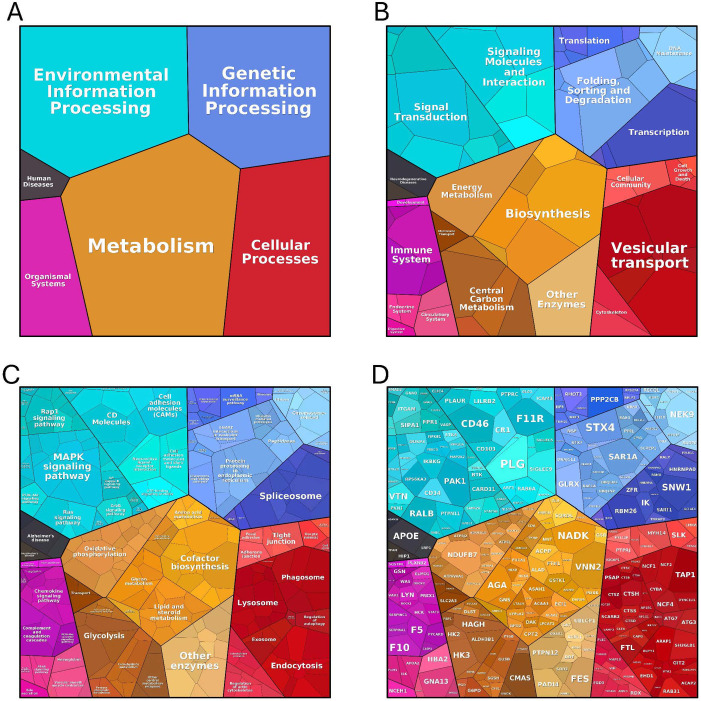
Proteomes of DMF- and DMSO-df-HL-60 cells. **Protein abundances are represented by polygon size and grouped by functional categories**. **A**: Broad functional categories reflect the overall functional organization of the proteome. **B**: Pathways involved in cellular adaptations that support differentiation. **C**: Enriched pathways critical for cellular functions and processes. **D**: Highly abundant genes associated with key cellular activities.

Furthermore, we used ProteoMap to examine the roles of individual proteins in key pathways and processes. The proteins are represented as polygons, with sizes reflecting fold changes in abundance, and functionally related proteins are grouped closely for clarity. ProteoMap figures highlight the differences in protein expression between untreated HL-60 cells and DMSO- or DMF-df-HL-60 cells. Each color represents distinct pathways or processes, and polygon sizes correspond to protein fold changes. The proteins identified in df-HL-60 cells were involved in metabolism, genetic and environmental information processing, and cellular organization, as shown in (Fig 12). Significant changes were observed in the environmental information processing category, with enriched proteins in signal transduction pathways such as Mitogen-Activated Protein Kinase (MAPK), Ras, and Rap1 signaling, which regulate proliferation, differentiation, and survival. The proteins related to cell adhesion molecules (CAMs) and CD molecules were also enriched, indicating enhanced immune signaling and cellular communication in differentiated HL-60 cells. These results suggest that DMF- or DMSO-df-HL-60 cells exhibit increased readiness to process environmental cues, a hallmark of effective differentiation.

### 3.10. Protein abundance for HL-60 cell differentiation markers

**Fig 13:**
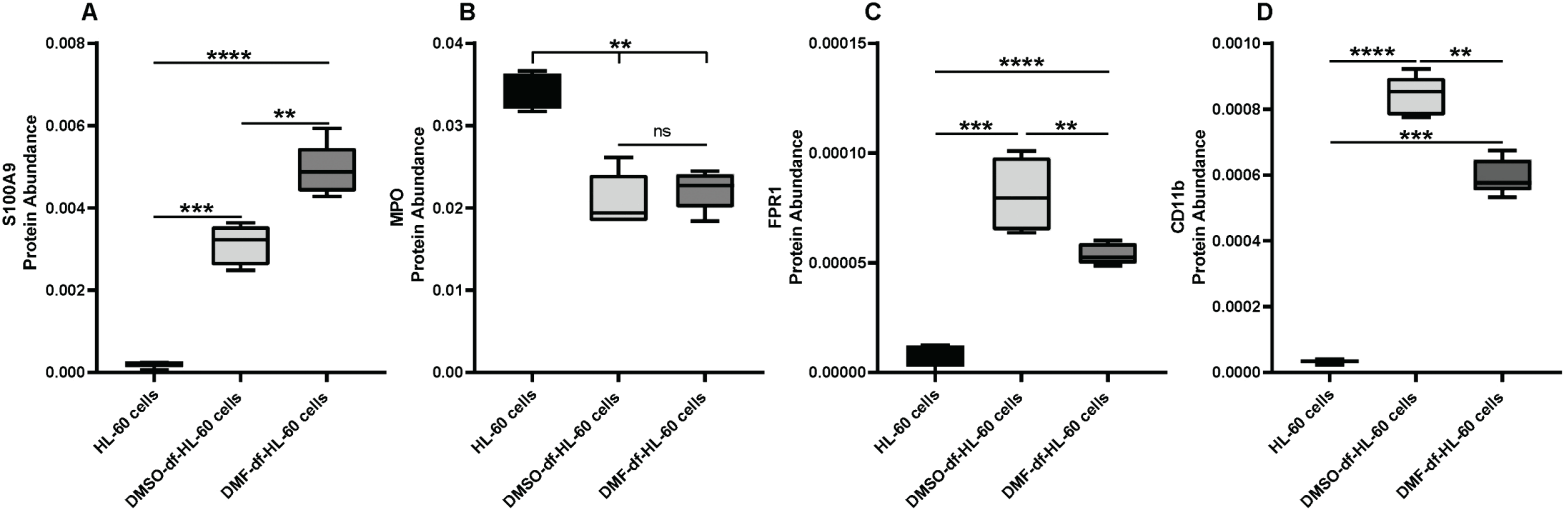
Protein abundance for neutrophil-like function markers in the differentiated HL-60 cells: A: S100A9 protein abundance, associated with key neutrophil functions, was significantly upregulated in differentiated HL-60 cells, with higher expression in DMF-treated cells compared to DMSO-treated cells. **B**: MPO protein amount, which decreases during differentiation, was reduced in both DMSO- and DMF-treated cells. **C**: FPR1 protein abundance, a late marker of granulocyte differentiation, significantly increased in HL-60 cells differentiated with DMSO or DMF compared to undifferentiated HL-60 cells. The protein expression levels were higher in DMSO-treated cells. **D**: The amount of CD11b protein, an early marker of neutrophil differentiation, significantly increased in HL-60 cells differentiated with DMSO or DMF compared to undifferentiated HL-60 cells. The protein expression levels were higher in DMSO-treated cells. Data are presented as mean ± SD from five independent experiments (n = 5). Statistical analysis was performed using one-way ANOVA with significance. (**P < 0.01 ***P < 0.001, ****P < 0.0001).

To assess neutrophil-like differentiation after five days of the differentiation process, we analyzed the protein abundance of key functional markers in untreated and differentiated HL-60 cells, as shown in Fig 12. Our results indicate a significantly high abundance in both early- and late-stage differentiation markers, including Integrin alpha M (CD11b) and N-formyl peptide receptor 1 (FPR1), in DMSO- and DMF-df-HL-60 cells, with DMSO-df-HL-60 cells showing a higher amount of protein as shown in Fig 13 (C & D). Furthermore, we assessed S100 Calcium-Binding Protein A9 (S100A9), a critical regulator of neutrophil function; the result showed a drastic increase in abundance in both differentiated groups compared to untreated HL-60 cells. DMF-df-HL-60 cells, however, demonstrated a significantly higher S100A9 abundance than their DMSO-treated counterparts, suggesting differential regulation of neutrophil-related proteins depending on the differentiation agent used, as depicted in Fig 13 (A). To further confirm differentiation, we also assessed the amount of MPO as an indicator of neutrophil maturation. Our results revealed a drastic decrease in MPO abundance in both DMSO- and DMF-df -HL-60 cells, as shown in Fig 13 (B). This decrease is consistent with the MPO activity and MPO protein expression data.

## 4. Discussion

The differentiation of HL-60 cells into neutrophil-like cells (df-HL-60) involves significant cellular and molecular changes, transforming these cells into functional models for studying neutrophil biology. Induced by agents such as DMSO or DMF, differentiation activates the expression of genes critical for neutrophil maturation, including those encoding granule proteins like MPO and neutrophil elastase [19,25–27]. These two agents are well-established for inducing granulocytic differentiation in HL-60 cells, activating distinct pathways and processes, leading to unique cellular adaptations.

Previous studies have investigated the proteomic changes during granulocytic differentiation of HL-60 cells induced by DMSO or ATRA, highlighting their role in activating molecular pathways essential for differentiation [42]. However, the proteomic changes during the DMF-induced differentiation of HL-60 cells remain underexplored, and a comprehensive proteomic analysis comparing the effects of DMSO and DMF has not been previously conducted. This study addresses this knowledge gap by evaluating the differentiation induced by DMF by not only assessing the functionality of df-HL-60 but also characterizing the changes in protein expression during DMF-induced differentiation in comparison with the effects of well-established DMSO.

### 4.1. Proteomic insights into differentiation

We found major alterations in protein expression patterns in the DMSO- and DMF-generated df-HL-60 proteomic profiles, which we attribute to important processes related to neutrophil function and development. The differences in protein abundance between the DMSO, DMF, and untreated groups were shown by a novel tri-plot analysis, revealing that the proteome profiles of the two treatments were more similar. Neutrophil degranulation and Rho GTPase signaling, were among the pathways in which the two distinct groups shared proteins in abundance. Additionally, both groups showed activation of mechanisms such as TLR cascade, receptor tyrosine kinase signaling, and phagocyte ROS and RNS production. While demonstrating the similar impact of DMSO and DMF in df-HL-60 cells, these results highlight the involvement of these pathways in promoting neutrophil differentiation and maturation. In addition, the proteins involved in signal transduction pathways that control proliferation, differentiation, and survival were found to be enriched using preotemap analysis. The proteins involved in signal transduction pathways regulating proliferation, differentiation, and survival were enriched, with the MAPK pathway being the most abundant. The ERK and p38 of MAPK are known to be involved in the differentiation and function of DMSO-df- HL-60 cells, influencing endocytosis, chemotaxis, and cytokine production [43–45]. Our proteomics findings suggest potential involvement of MAPK/Ras-associated signaling during DMF-induced HL-60 differentiation; however, this requires functional validation. Since MAPK signaling has been mechanistically validated in DMSO-induced differentiation [45], their role in DMF-mediated differentiation remains to be confirmed.

### 4.2. Transition to a neutrophil-like phenotype

During the differentiation of HL-60 cells induced by DMSO and DMF, the cells transition from a proliferative to a specialized neutrophil-like phenotype, resulting in decreased proliferation. The trypan blue exclusion tests showed a reduction in cell numbers by both DMF and DMSO by day five. Our findings reveal a critical distinction in how DMSO and DMF impact HL-60 cell proliferation, despite both being established differentiating agents. While both 1.25% DMSO and 70 mM DMF (≈ 0.5%) successfully differentiated the cells, their effects on proliferation kinetics were divergent. DMSO treatment led to a rapid and significant reduction in cell numbers, with a continued decline over time, suggesting not only strong inhibition of cell proliferation but also potential cell loss or cytotoxicity. This aggressive effect implies that DMSO’s influence on the cell population extends beyond simple differentiation, possibly inducing stress or death pathways that could confound studies primarily focused on differentiation markers. In contrast, DMF treatment resulted in a stable proliferation arrest, where cell counts plateaued without further decline, indicative of cells exiting the cell cycle and possibly progressing to differentiation. This suggests that DMF provides a cleaner model for studying differentiation mechanisms, as it promotes maturation without significant cell death. This differential impact on cell viability and proliferation kinetics underscores the importance of carefully selecting the appropriate differentiating agent based on specific experimental objectives, as the underlying cellular responses to DMSO and DMF are markedly distinct. Our proteomic results showed a significantly lower abundance of cell cycle regulator proteins, including cyclin-dependent kinases (CDK1 and CDK11B) and the Minichromosome Maintenance Complex (MCM2–7), essential for cell cycle progression, as shown in S1 Table.

### 4.3. Markers of HL-60 differentiation

The differentiation of promyelocytic HL-60 cells to neutrophil-like cells was assessed using early- and late-stage markers. CD11b, an early-stage marker regulating neutrophil adhesion and migration, was significantly more expressed in DMSO-treated cells (85%) compared to DMF-treated cells (65%) after five days (Fig 3). Our proteomic outcomes corroborated these findings, showing a marked abundance of CD11b protein in DMSO-treated cells compared to DMF-treated cells, as shown in Fig 12. The late-stage differentiation markers, such as FPR1, which enhanced chemotactic responses and the oxidative burst, exhibited significant protein abundance as depicted in Fig 12. The abundance of FPR1 was significant in DMSO-df-HL60 cells compared to the DMF-df-HL60 cells, indicating that HL-60 cells had acquired neutrophil-like characteristics by five days.

Moreover, we measured MPO expression and activity, as it is the hallmark of neutrophils, where MPO catalyzes the formation of oxidants critical for antimicrobial activity [46,47]. Over the five-day differentiation period, MPO levels progressively decreased in both groups, with DMSO-df-HL60 cells showing a more rapid reduction, as shown in Figs 6 & 7, which is consistent with our proteomic result, as shown in Fig 12. This decrease aligns with previous findings that differentiation reduces MPO synthesis as cells transition to a neutrophil-like state [48,49]. This finding is consistent with a previous study, which reported that while MPO activity decreases during the differentiation of HL-60 cells by DMSO, it remains higher than that observed in primary human neutrophils [50].

The oxidative burst is another functional marker indicative of granulocytic differentiation [32, 51, 52]. To thoroughly characterize ROS production, we strategically used a dual-assay approach: the established NBT assay, complemented via the advanced technique of EPR spectroscopy. This integration allowed for comprehensive validation of our findings.

Both methodologies showed a significant increase in superoxide production in both DMSO- and DMF-treated HL-60 cells when compared to their untreated counterparts, thereby confirming the successful induction of oxidative burst. Notably, the EPR spectroscopy, serving as a powerful new tool for precise radical detection, revealed that the DMF-treated cells exhibited a more distinctly defined superoxide spectrum than the DMSO-treated cells. This more refined spectral output from EPR is potentially reflective of a heightened and more consistent flux of radical formation within the DMF-treated population, providing nuanced insight into the robust ROS generation observed across both assays. [35, 39, 40] NOX and MPO are the key components of ROS production that play a central role in neutrophil function, such as NET formation, phagocytosis, and degranulation, which contribute to antimicrobial activity [32, 51, 52]. We confirmed that proteomic data supported this, showing a high protein abundance of NOX components, including. CYBB (gp91phox), the catalytic subunit of NOX [53], exhibited higher abundance in DMF-treated cells compared to DMSO-treated cells, suggesting a potentially higher capacity for ROS generation in DMF-treated cells.

### 4.4. Neutrophil granule formation in df-HL-60

The functional enrichment analysis of unstimulated df-HL-60 cells using Metascape revealed the upregulation of proteins associated with granule mobilization and degranulation, highlighting the functional maturation of granules during differentiation. The key azurophilic granule components, such as cathepsin D, was highly abundant in both DMF- and DMSO-treated groups. Cathepsin D is a major proteolytic enzyme in lysosomes and phagosomes [54,55]. It plays a crucial role in the breakdown of material within phagocytes in neutrophils and is essential for the degradation of engulfed particles during phagosome maturation and is also involved in immune responses and cell death regulation [54,55]. Furthermore, cytochrome b558 subunits (CYBA and CYBB) also showed high abundance in both groups compared to untreated cells, signifying the development of a robust NOX complex crucial for the generation of ROS and antimicrobial defense [44]. The secondary granule markers, such as Lysosome-associated membrane glycoprotein 2 (LAMP2), play a crucial role in neutrophil function by regulating phagosomal maturation and fusion with lysosomes, allowing for the degradation of engulfed material by lysosomal enzymes [56,57]. The abundance of LAMP2 was also considerably higher in both groups compared to untreated cells. The tertiary granule markers, including Rab27A, were dramatically abundant in both groups. Rab27A is the predominant Rab27 isoform present in human neutrophils and plays a major role in the exocytic machinery, modulating the secretion of tertiary granules [58]. It also plays an essential role in NET formation by regulating ROS production in neutrophils [59]. The upregulation of tertiary granule-associated proteins further suggests that these granules are readily mobilized and functional in df-HL-60 cells.

We found through proteomic analysis that the levels of calcium-binding proteins S100A8 and S100A9 during HL-60 cell differentiation induced by DMSO and DMF were significantly higher than in untreated cells, with a high abundance observed in DMF-treated cells, as shown in Fig 12, which may explain why DMF-df-HL-60 cells are identified as a suitable model for studying NETs [60]. Previous studies have shown that S100A9 is highly expressed in HL-60 cells differentiated with ATRA [61,62]. Our findings demonstrated that the S100A8/A9 complex was markedly higher in DMSO- and DMF-df-HL-60 cells. S100A9 has been reported to induce differentiation and growth arrest of acute myeloid leukemia (AML) cells via Toll-like receptor-4 (TLR4), while S100A8 regulates S100A9 activity and sustains the AML immature phenotype [62]. This complex regulates key neutrophil functions such as cytokine secretion, NOX activation, and degranulation [63]. Their substantial abundance during differentiation suggests a key role in granule trafficking and stabilization of cytoskeletal dynamics [63]. The higher levels of S100A8 and S100A9 proteins in DMF-df-HL-60 cells align with enhanced activation of signaling pathways critical for neutrophil functionality, highlighting their potential as targets for effective differentiation of HL-60 cells. Further studies are needed to validate the proteomic finding of S100A9/A8 upregulation using an independent method.

### 4.5. Differences in the proteomic profiles with the various differentiation agents

To contextualize the DMF-df-HL-60 proteomic profile, we compared our findings with published datasets from ATRA-induced granulocytic/neutrophil-like HL-60 models. Overall, DMF differentiation shows key features of granulocytic maturation reported for ATRA, including upregulation of the neutrophil surface integrin CD11b (ITGAM) and induction of immune and inflammatory effector programs involving proteins such as PAD4, S100A8/S100A9, SERPINB1, and neutrophil elastase (ELANE) [64]. In parallel, reduction in cell cycle-associated proteins was observed, marked by reduced abundance of proteins involved in DNA replication and repair, including MCM2–7, DDX18, DDX21, PARP1, and RPA1 [64]. A distinct difference was observed in MPO expression across differentiation models. MPO, a hallmark enzyme of primary azurophilic granules, was markedly reduced in both DMF- and DMSO-df-HL-60 cells. Despite this reduction, previous research has demonstrated that MPO levels in DMSO-df-HL-60 cells remained higher than those observed in primary human neutrophils [50]. Interestingly, MPO expression was also reported to be increased in ATRA-df- HL-60 cells [65]

Furthermore, a previous report on ATRA-df- HL-60 cells showed that proteinase 3 (PRTN3) can act as an autocrine inhibitor of granulocytic maturation, the decreased PRTN3 levels observed in our DMF-df- HL-60 cells may reflect reduced inhibitory signaling and support progression toward a more neutrophil-like differentiated state [66]. While cathepsin G (CTSG) was identified to be upregulated during ATRA-driven terminal differentiation, it was reduced in both DMF and DMSO conditions [65]. Notably, oxidative effector pathways further distinguished the DMF model at the proteomic level, as DMF-dHL-60 cells showed coordinated upregulation of multiple components of the NADPH oxidase (NOX2) system, consistent with priming of a ROS-generating neutrophil-like phenotype. In contrast, previously published ATRA-based proteomic studies have generally reported limited detection of NADPH oxidase subunits, most commonly identifying NCF1 (p47phox). Importantly, non-proteomic functional studies have demonstrated NADPH oxidase-dependent oxidative metabolism during ATRA-driven granulocytic differentiation, confirming NOX2 functionality despite incomplete proteomic recovery [67]. These findings suggest that differences in the proteomic profiles of the various differentiating agents could dictate specific pathways and the unique mechanisms of differentiation by each agent. Following this, we tested the ability of both models to release NETs as a key neutrophil effector function. NET is an antimicrobial defense mechanism in which neutrophils release extracellular chromatin structures to trap and neutralize pathogens. Sytox Green assay showed robust NET release in the DMF-differentiated model, as shown in Fig 8 supporting its efficiency as a reliable *in vitro* platform for studying NET formation.

## 5. Conclusion

This study provides the first comprehensive proteomic characterization of DMF-induced HL-60 granulocytic differentiation and places it in direct comparison with the widely used DMSO-df-cell model. Despite differences in solvent identity and differentiation kinetics, DMF produced phenotypic, functional, and proteomic signatures consistent with neutrophil-like maturation and largely overlapping with those observed in DMSO-df- cells. Notably, DMF achieved this neutrophil-like phenotype at a lower working concentration with improved proliferation and reduced cytotoxicity, offering practical advantages for downstream functional studies. Consistent with proteomic evidence of enhanced inflammatory and oxidative programs, DMF-df- HL-60 cells also exhibited robust NET formation, supporting its suitability as a reliable in vitro platform for investigating NETosis and ROS-dependent neutrophil biology.

## Supporting information

S1 Fig Uncropped immunoblots to support the data presented in Fig. 7 for MPO and β-actin (loading control) detection. (PDF)

S1 Table Complete detailed list of protein expression from DMSO and DMF treated cells. The abundance ratios and preprocessed data were subsequently used for gene ontology analysis.
(XLSX)
