# Supplementary material for: Unraveling dimethylformamide-induced neutrophilic differentiation in HL-60 cells: A proteomic and functional comparison with dimethyl sulfoxide
Source: PLoS One. 2026 May 13;21(5):e0348783. doi: 10.1371/journal.pone.0348783 (PMC13170858; doi:10.1371/journal.pone.0348783)
Supplement: S1 Fig — (PDF) [file pone.0348783.s001.pdf]

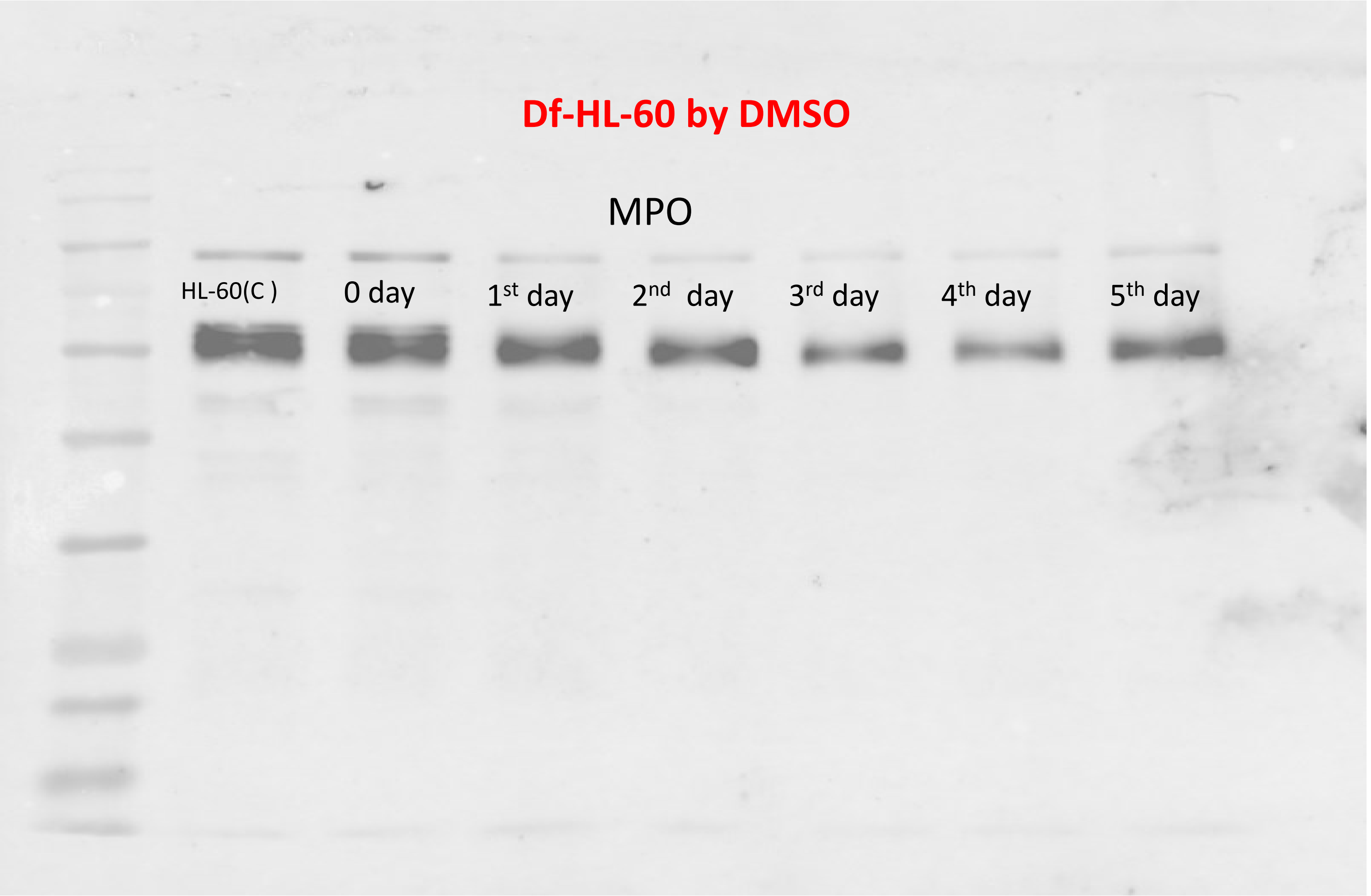

0 DAY means I treat the cells with DMSO and lysate them in the same day.

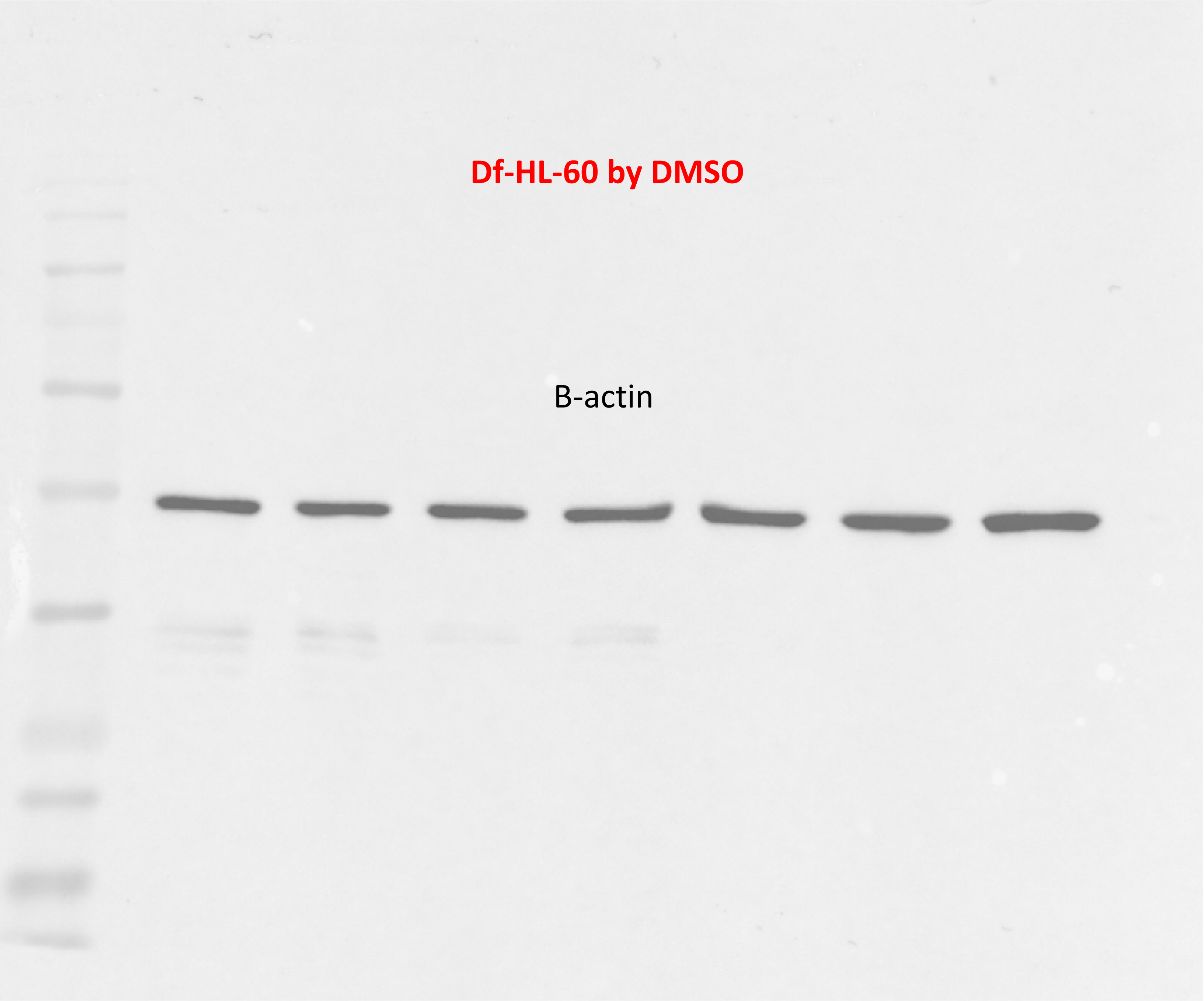
Df-HL-60 by DMSO
B-actin

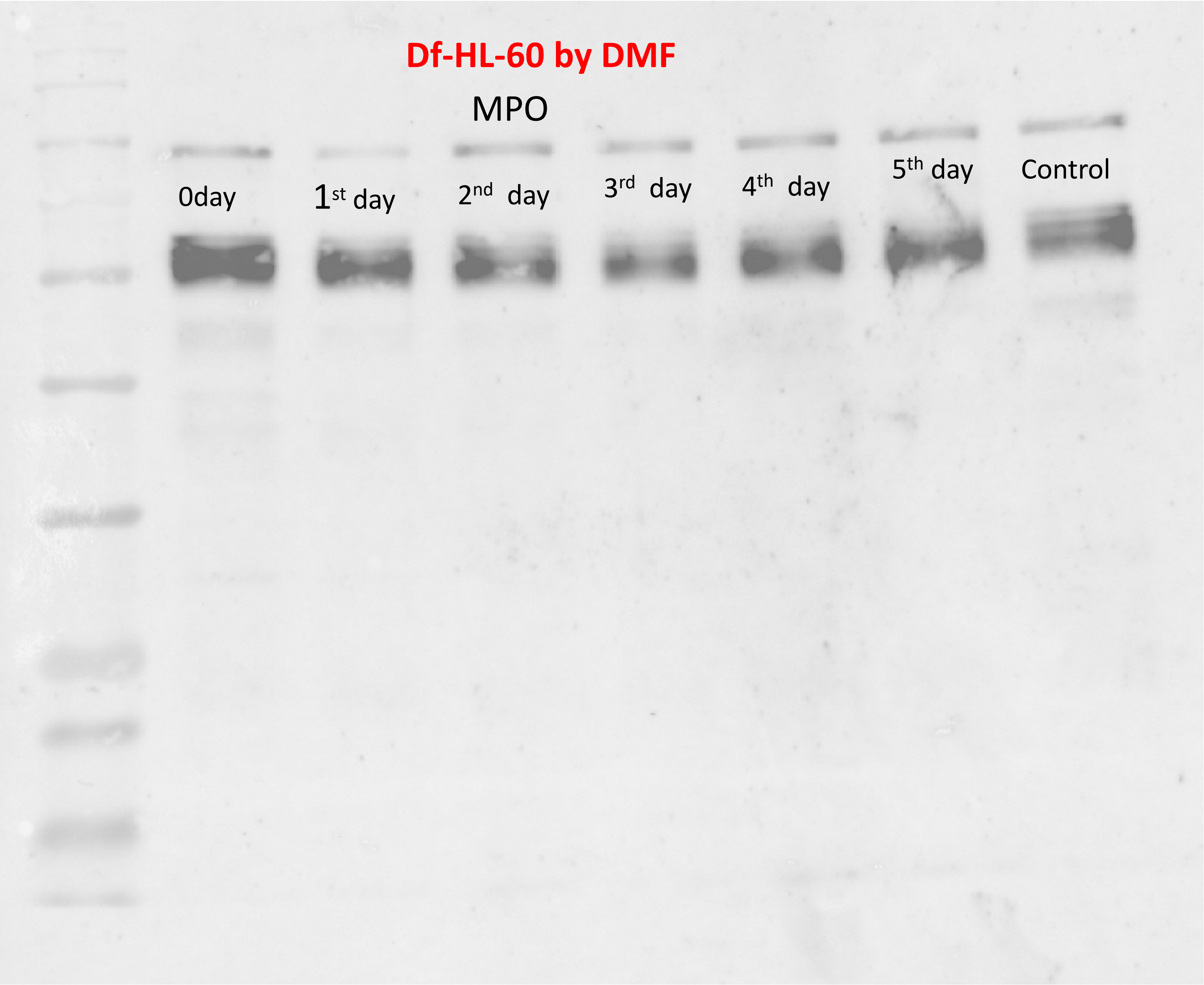
Df-HL-60 by DMF
MPO
0day
1st day
2nd day
3rd day
4th day
5th day
Control

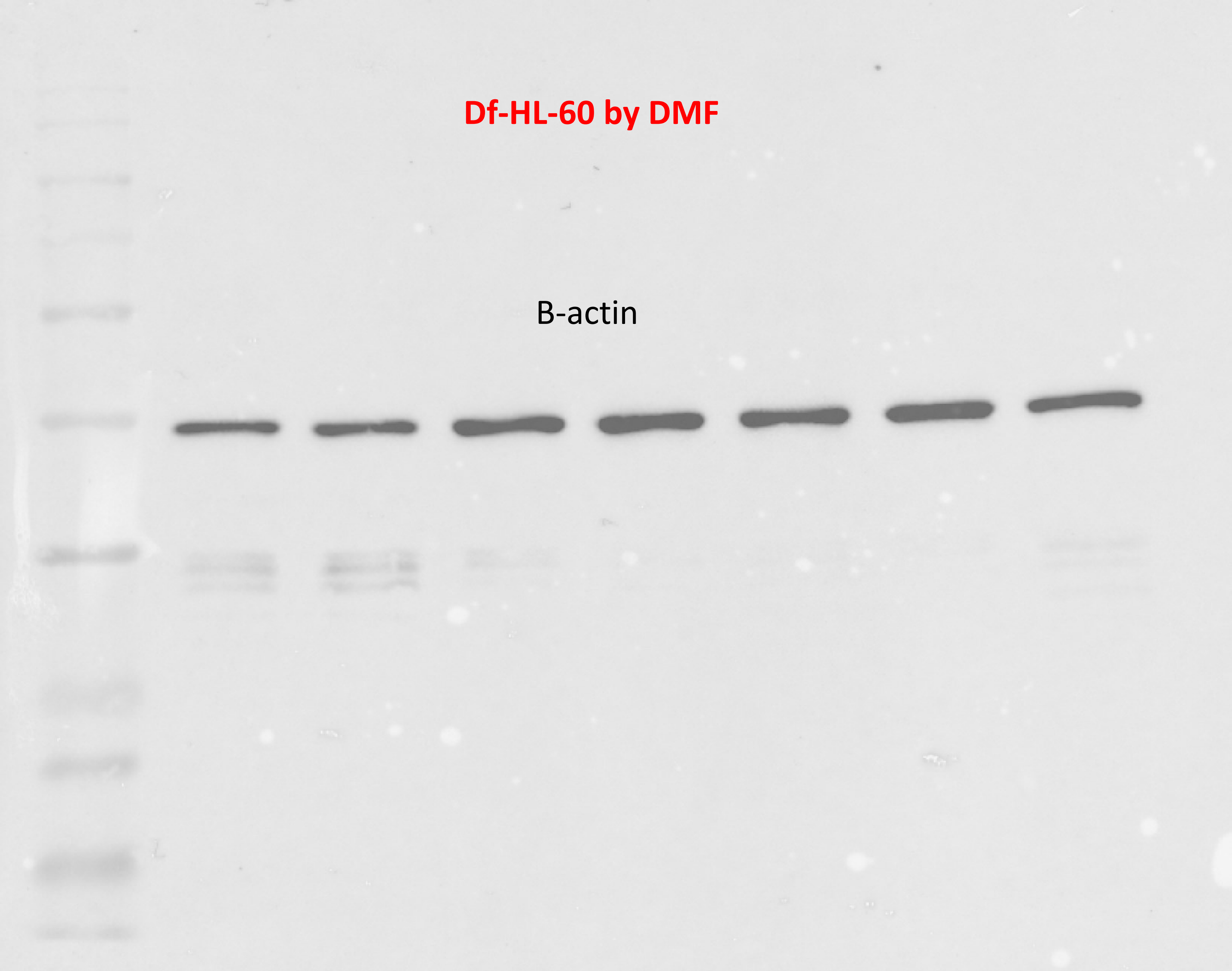
Df-HL-60 by DMF
B-actin
